# Wasp-Waist Interactions in the North Sea Ecosystem

**DOI:** 10.1371/journal.pone.0022729

**Published:** 2011-07-28

**Authors:** Per Fauchald, Henrik Skov, Mette Skern-Mauritzen, David Johns, Torkild Tveraa

**Affiliations:** 1 Department of Arctic Ecology, Norwegian Institute for Nature Research (NINA), Fram Centre, Tromsø, Norway; 2 DHI, Hørsholm, Denmark; 3 Institute of Marine Research, Bergen, Norway; 4 Sir Alister Hardy Foundation for Ocean Science (SAHFOS), Plymouth, United Kingdom; National Institute of Water & Atmospheric Research, New Zealand

## Abstract

**Background:**

In a “wasp-waist” ecosystem, an intermediate trophic level is expected to control the abundance of predators through a bottom-up interaction and the abundance of prey through a top-down interaction. Previous studies suggest that the North Sea is mainly governed by bottom-up interactions driven by climate perturbations. However, few studies have investigated the importance of the intermediate trophic level occupied by small pelagic fishes.

**Methodology/Principal Findings:**

We investigated the numeric interactions among 10 species of seabirds, two species of pelagic fish and four groups of zooplankton in the North Sea using decadal-scale databases. Linear models were used to relate the time series of zooplankton and seabirds to the time series of pelagic fish. Seabirds were positively related to herring (*Clupea harengus*), suggesting a bottom-up interaction. Two groups of zooplankton; *Calanus helgolandicus* and krill were negatively related to sprat (*Sprattus sprattus*) and herring respectively, suggesting top-down interactions. In addition, we found positive relationships among the zooplankton groups. *Para/pseudocalanus* was positively related to *C. helgolandicus* and *C. finmarchicus* was positively related to krill.

**Conclusion/Significance:**

Our results indicate that herring was important in regulating the abundance of seabirds through a bottom-up interaction and that herring and sprat were important in regulating zooplankton through top-down interactions. We suggest that the positive relationships among zooplankton groups were due to selective foraging and switching in the two clupeid fishes. Our results suggest that “wasp-waist” interactions might be more important in the North Sea than previously anticipated. Fluctuations in the populations of pelagic fish due to harvesting and depletion of their predators might accordingly have profound consequences for ecosystem dynamics through trophic cascades.

## Introduction

The ongoing scientific debate of whether marine ecosystems are influenced by top-down or bottom-up processes is fundamental for understanding how drivers of change affect ecosystem dynamics. According to the bottom-up view, climate change is the major process behind recent changes in marine ecosystems [1]–[3]. The top-down view, on the other hand, holds that shifts in marine ecosystems are mainly due to overfishing of top predators [4], [5]. Perturbations at the base of the food web will, in an ecosystem governed by bottom-up processes, propagate upward through the food web. As long as the species composition is kept intact, the system is expected to show predictable, donor controlled responses to perturbations, and to return to its prior state when the external perturbation ceases [6], [7]. Because a perturbation at the top is unlikely to cascade down the food web, such systems are relatively robust with respect to harvesting [8], [9]. While bottom-up processes generally enhance ecosystem resilience, top-down interactions may result in trophic cascades and internal positive feedbacks within the food web [5], [10]. An ecosystem subject to strong top-down forcing is therefore expected to exhibit several alternative stable states under the same external conditions. A perturbation of such systems may be followed by a reorganization of the trophic structure resulting in a non-linear ecosystem shift [11].

Marine pelagic ecosystems in upwelling and coastal areas are often characterized by highly diverse upper and lower trophic levels and a less diverse intermediate level [12], [13]. The upper level consists of predatory fish, seabirds and sea mammals while the lower trophic levels consist of a diverse assemblage of phytoplankton and zooplankton species. The intermediate level that links zooplankton and top-predators is usually occupied by a few dominating pelagic forage fish species that has been suggested to control the upper trophic level through a bottom-up interaction and the lower trophic level through a top-down interaction [12]. Because of the striking difference in the diversity among the three upper trophic levels, this particular system has been termed a “wasp-waist” system [12]. Cod is a major top-predator in northern shelf ecosystems [14]. In ecosystems such as the Baltic Sea and the Scotian Shelf, intensive harvesting and the subsequent decline in cod abundance has been followed by a marked increase in the populations of pelagic forage fishes [5], [15]. Pelagic forage fishes are predators and competitors to the early life stages of cod, and they might accordingly prevent the recovery of one of their major predators [15]–[18]. Such predator-prey role reversals generate internal positive feedbacks which again promote ecosystem hysteresis [18]. A large population of cod will, according to this hypothesis, secure its own recruitment by controlling the abundance of forage fish and thus keep the system in a cod dominated state. Conversely, high abundance of forage fish will reduce the recruitment of cod and thus keep the system in a forage fish dominated state. Selective fishing on the dominant group, will perturb the system, and might “push it” to the alternate state. If the pelagic forage fish affect the abundance of zooplankton through a top-down effect and/or other predator groups through a bottom-up effect, selective fishing could potentially result in a trophic reorganization of the ecosystem [5], [15], [17].

The North Sea is one of the most heavily fished marine ecosystems in the world, resulting in a fishing mortality that currently is above what is considered to be sustainable for many of the exploited stocks [19]. Despite this massive human perturbation, recent changes in the plankton community has largely been related to climate, particularly changes in the strength of westerly winds that affect local climate, as well as the inflow of oceanic water into this semi-closed ocean basin [2], [20]–[23]. An abrupt change in climate in the 1980s was associated with a shift in the recruitment of a number of fish species and changes in the plankton community, suggesting that a climate driven regime shift took place in this period [2], [22], [24]. Thus, although some top-down forced changes have been suggested [25]–[28], a majority of studies suggest that the North Sea system is mainly driven by bottom-up forces through climate [1], [3], [23]. Based on analyses of a 30-year time series of production and consumption in the fish food web of the North Sea, [26] it is suggested that bottom-up forces mainly control the dynamics of the pelagic food webs, while top-down forces control the benthic food webs.

Although pelagic forage fishes are expected to play a central role in wasp-waist ecosystems [12], [13], and in particular in northern shelf ecosystems [5], [15], [17], few studies from the North Sea have considered the possible top-down effect from pelagic forage fish on the recruitment of predator fishes and the abundance of zooplankton (but see [25]). Recently, [18] found a negative relationship between the abundance of herring (*Clupea harengus*) and the recruitment of cod, suggesting that predator-prey role reversal could promote ecosystem hysteresis in the North Sea. He suggested that the current intensive harvesting of both herring and cod prevent the system from settling in a stable state, and that the system, as a consequence, fluctuates between two quasi-stable states. In the present study, we investigate how the large fluctuations in the abundance of clupeid forage fish might affect the zooplankton community through top-down interactions and the abundance of seabirds through bottom-up interactions.

Key predation from e.g. dominant pelagic fish might have a range of subtle effects on the prey community as predation might affect the interspecific interactions among the prey species [29], [30]. This is particularly important when the predator is selective and switches between different prey species depending on their relative abundance [30], [31]. Clupeid fishes are strongly selective with respect to the size and availability of their zooplankton prey [32], [33]. This selectivity is related to two distinct modes of foraging; filter feeding for small copepods and visual predation on larger copepods and krill [34]. When the abundance of the large prey species drops below a certain level, the clupeid fishes might change their feeding behavior from particulate visual predation to filter feeding on smaller food items [31]. Under high abundance, the preferred prey will accordingly protect the less preferred prey from predation. This could potentially reduce the possibility of competitive exclusion [30]. However, it might also affect the numerical relationship between the two prey groups. This is because the abundance of the preferred prey will reflect both the abundance of predators and the protection of the less preferred prey from predation. The result will be a strong positive relationship between the two prey categories.

In this study, we investigate the long-term numerical relationships between ten pelagic seabird species, two species of clupeid fishes, and four groups of zooplankton from the North Sea. Predation on zooplankton is expected to be strongest during spring and summer [33]. Because we wanted to investigate the numeric relationships after the major consumption had taken place, we decided to use the winter abundance of zooplankton in the analyses. This measure should be a result of both the production and consumption during the previous spring and summer. If consumption from clupeid fishes is important, we expected to find negative relationships between the abundance of fish and the winter abundance of zooplankton. Outside the breeding season, seabirds are free to roam of large areas in the search for food. The winter abundance of seabirds in the North Sea will therefore to some degree reflect the relative profitability of the North Sea as a winter area. We expected accordingly that the abundance of wintering seabirds in the North Sea should be responsive to the abundance of prey. If the abundance of clupeid fishes is important, we expected to find positive relationships between the abundance of seabirds and fish.

## Results

**Figure 1:**
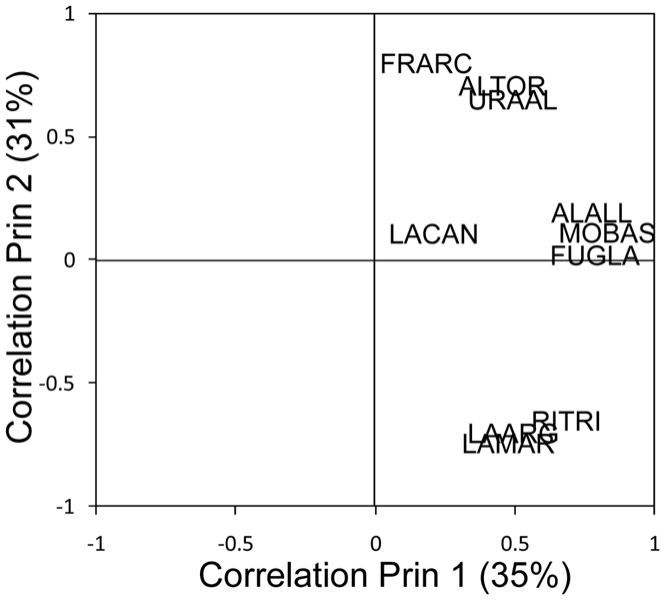
Principal component analysis (PCA) of yearly winter abundance of 10 seabird species in the North Sea. The plot shows the Pearson's correlation coefficients between the abundance estimates and Principal component 1 (Prin 1) and Principal component 2 (Prin 2). Percentage of total variance explained by the two principal components is indicated. Abundance estimates were log_10_ transformed prior to the analyses. Species are: Little auk, *Alle alle* (ALALL); razorbill, *Alca torda* (ALTOR); northern fulmar, *Fulmarus glacialis* (FUGLA); Atlantic puffin, *Fratercula arctica* (FRARC); herring gull, *Larus argentatus* (LAARG); common gull, *Larus canus* (LACAN); great black-backed gull, *Larus marinus* (LAMAR); northern gannet, *Sula bassana* (MOBAS); black-legged kittiwake, *Rissa tridactyla* (RITRI) and common murre, *Uria aalge* (URAAL).

An initial screening of the data indicated that the winter abundance of the different seabird species co-varied among years (see Fig. S1). A Principal Component Analysis (PCA) supported this observation as all species were positively associated with the first axis (Prin1), explaining 35% of the variance in the abundance estimates (Fig. 1). We therefore used the yearly score of Prin1 as a measure of total seabird abundance. Prin2 of the PCA explained another 31% of the variance in the abundance estimates. Contrary to Prin1, Prin2 explained the difference in dynamics among the species. Specifically, it discriminated between the different dynamics of some gulls (kittiwake, herring gull and great black-backed gull) and auks (Atlantic puffin, razorbill and common murre). No significant relationships were found between Prin2 and the clupeid fishes or sea surface temperature (SST).

**Figure 2:**
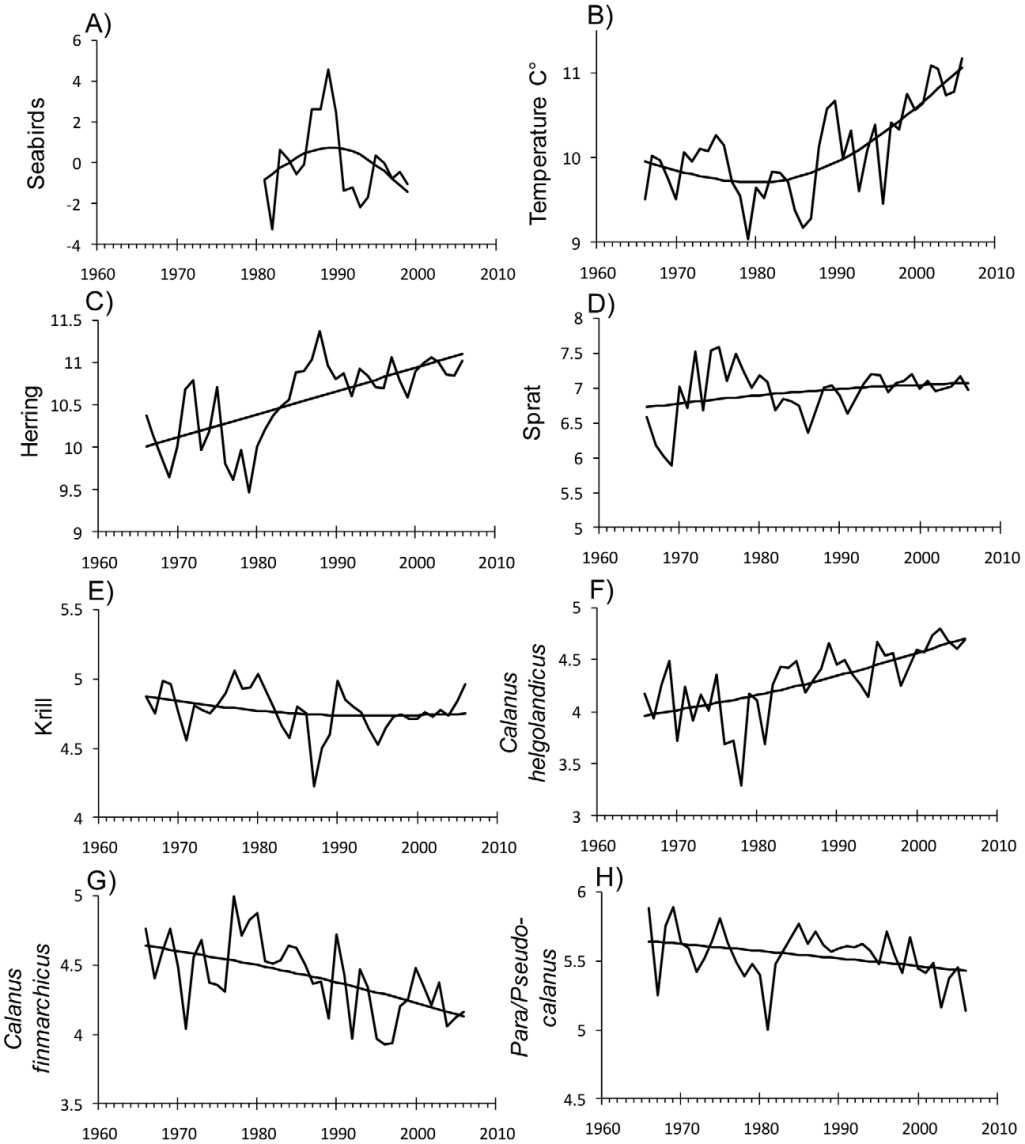
Time series of A) seabirds, B) sea surface temperature C–D) clupeids and E–H) zooplankton in the North Sea. Seabirds, clupeids and zooplankton are estimated winter abundance, sea surface temperature is yearly average temperature. Seabird is the first principal component of seabird abundance. Abundance of zooplankton and clupeids are log_10_-transformed. Lines are predicted trends from GAM analyses.

The time series with trends fitted by GAM functions are shown in Fig. 2. The abundances of the two pelagic fish species were poorly correlated (original data; r = 0.16, detrended data; r = −0.06). Linear models of seabird abundance and the four different zooplankton groups were deployed with respect to SST, clupeids and interspecific interactions (zooplankton only). For analyses of seabirds the sample size was 19 years (1981–1999), and for zooplankton the sample size was 41 years (1966–2006).

The estimates from the final models (after model selection) are shown in Table 1. Analyses of original and detrended data are shown for comparison. Detrending had a large impact on the estimated contribution from SST. Analyses on the original data showed strong relationships between SST and all four groups of zooplankton. After detrending however, SST was only present as a significant term in the model of *Calanus helgolandicus*. Thus, the relationship between SST and zooplankton was primarily a consequence of similar trends in the dataseries. For *C. helgolandicus*, a positive relationship with herring was found in the original data, however this relationship disappeared and a positive relationship with *Para/Pseudocalanus* appeared after detrending. The other trophic and interspecific relationships were robust with respect to detrending as they appeared as significant terms in both groups of models. The positive relationship between herring and *C. helgolandicus* in the original data is hard to explain. Since this relationship disappeared after detrending, we suggest it was spurious and due to similar trends in the two dataseries.

**Table 1:** Estimates from linear models relating yearly abundance of seabirds and zooplankton to sea surface temperature (SST), abundance of clupeid fishes (herring and sprat), the abundance of other species within the same trophic level (zooplankton only) and an autoregressive term (AR-1).

	SST	Herring	Sprat	Krill	*Calanus* *finmarchicus*	*Calanus helgolandicus*	*Para/pseudocalanus*	AR-1 (ρ)
Seabirds, n = 19								
Original data^a^	x	3.08*	x					0.52
Detrended data^a^	x	3.68**	x					x
Krill, n = 41								
Original data	0.14**	−0.17**	x		0.27**	x	x	x
Detrended data	x	−0.20**	x		0.26**	x	x	x
*Calanus finmarchicus*, n = 41								
Original data	−0.24**	x	x	0.90***		x	x	0.36
Detrended data	x	x	x	0.76***		x	x	x
*Calanus helgolandicus*, n = 41								
Original data	0.26**	0.36***	−0.30**	x	x		x	x
Detrended data	0.24*	x	−0.33***	x	x		0.58**	x
*Para/Pseudocalanus* spp., n = 41								
Original data	−0.20**	x	x	x	x	0.28**		0.40
Detrended data	x	x	x	x	x	0.32**		x

a Response lagged with one year,

*0.01<*P*<0.05,

**0.001<*P*<0.01,

***P<0.001.

‘x’ indicates removed terms according to the backward selection procedure.

**Figure 3:**
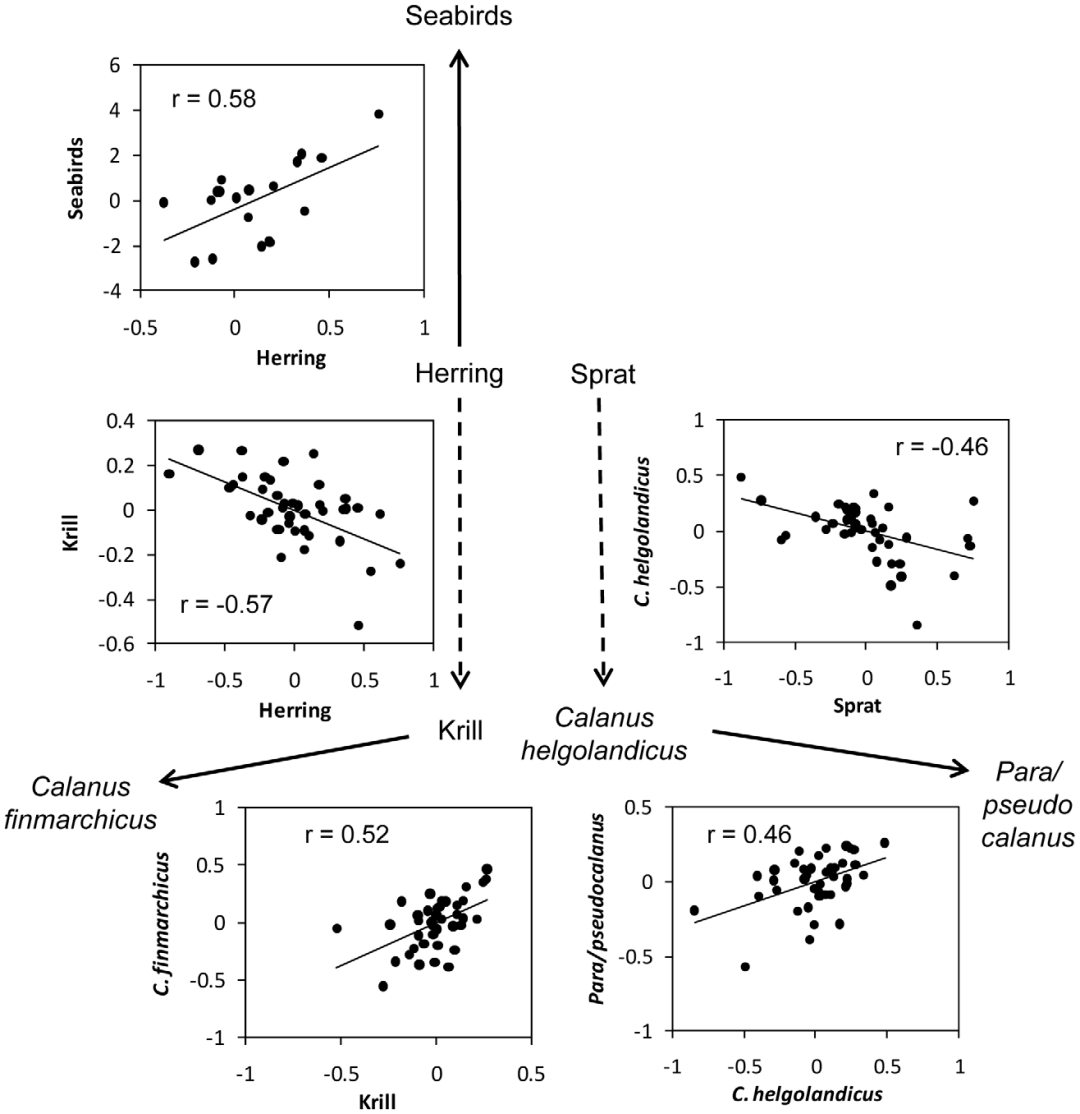
Trophic and interspecific relationships between the winter abundance of seabirds, clupeids and zooplankton in the North Sea. Plots show the relationships between detrended winter abundance of different species groups. *r* is Pearson's correlation coefficient. Only significant relationships from the model selection procedure are shown.

As expected, from a bottom-up perspective, the abundance of seabirds was positively related to the abundance of herring (Table 1, Fig. 3). However, no relationship was found between seabirds and sprat. Note that the relationship with herring was only present when the response of seabirds was lagged with one year. No significant relationships were found between seabirds and the covariates for unlagged data. For zooplankton, the picture was more complicated. In accordance with the top-down hypothesis, two species showed negative relationships with their predators; krill was negatively related to herring and *C. helgolandicus* was negatively related to sprat (Table 1, Fig. 3). For the two other zooplankton groups we found only weak relationships with the abundance of pelagic fish, but positive relationships with other zooplankton; *C. finmarchicus* was positively related to krill and *Para/Pseudocalanus* was positively related to *C. helgolandicus* (Table 1, Fig. 3). This is in accordance with a predator switching response. Since krill is the largest and possibly the most valuable prey item, *C. finmarchicus* is protected from herring predation when krill is abundant, resulting in a positive relationship between krill and *C. finmarchicus*. Similarly, *C. helgolandicus* is larger than *Para/Pseudocalanus*, and *Para/Pseudocalanus* would accordingly be protected from sprat predation when *C. helgolandicus* is abundant.

## Discussion

In a “wasp-waist” ecosystem an important intermediate trophic level is expected to control the abundance of predators through a bottom-up interaction and the abundance of prey through a top-down interaction [12]. Small pelagic schooling fishes such as herring and sprat, have been suggested to hold this position in northern coastal shelf ecosystems [13]. The present study supports these predictions for the North Sea ecosystem. From the bottom-up perspective, the abundance of different seabird species overwintering in the North Sea varied synchronously from year to year and was positively related to the abundance of herring. From the top-down perspective the abundance of zooplankton prey was inversely related to the abundance of herring and sprat. Comprehensive modeling of the North Sea ecosystem [35] identified clupeids together with sandeel as key consumers and important food items for predatory fish, sea mammals and seabirds, thus supporting the notion of theses species' important position in the food web. Moreover, simulations of different fishing regimes and fitting model output to historic dataseries, indicated that fishing was a major driver of the ecosystem [35], [36], suggesting that top-down interactions are indeed important in structuring the system. Recent findings suggest that selective fishing on cod or herring can push the North Sea ecosystem between a herring and a cod dominated state respectively [18]. The present study suggests that the resulting fluctuations in the stocks of clupeid fishes have pervasive effects on the seabird and zooplankton communities.

Due to low reproductive rates, the population response to changes in prey abundance is expected to be slow in seabirds [37]. Accordingly, the synchronous changes in the abundance of wintering seabirds probably do not reflect changes in population sizes, but might rather reflect the proportional use of the North Sea as a winter area [38]. Indeed the large year to year changes in the abundance of seabirds (cf. Fig. S1), indicate that they are highly responsive to changes in the ecosystem. Thus, contrary to more stationary predators such as cod, we expected a strong positive numeric response of seabirds to changes in their prey base. Both herring and sprat were expected to be important prey items for seabirds, and as expected, the results indicate that the North Sea was a profitable winter habitat for seabirds in years when herring was abundant. Contrary to our expectations, no significant relationship between seabirds and sprat was found. It should however be noted that the time series of seabirds was relatively short (19 years) making it less likely to find significant relationships. [38] suggested that the synchronous change in the winter abundance of different seabird species in the North Sea could be due to commensal foraging. Pelagic seabirds in the North Sea aggregate in multispecies feeding flocks where conspicuous species such as kittiwakes works as catalysts by discovering prey patches and diving auks make food available at the surface [39]. According to this hypothesis, the abundance of an important facilitating species such as the common murre [39] might be important in determining the profitability of the habitat for other species. The result would be synchronous changes in the winter abundance of different seabird species dictated by a few key species. Thus, our results can be explained by a combination of dynamics in prey abundance and commensal foraging. However, longer time series of seabird abundance, studies of winter habitat use and detailed studies of multispecies foraging flocks are needed to disentangle the importance of the different mechanisms involved.

Our results indicate that the two clupeid fishes had a large impact on the zooplankton community. The two fish species were related to two different groups of zooplankton. While krill was negatively related to herring, *Calanus helgolandicus* was negatively related to sprat. In addition, we found positive relationships between *C. finmachicus* and krill and between *Para/Pseudocalanus* and *C. helgolandicus*. Note that the largest species in each of these pairs (krill and *C. helgolandicus* respectively) were negatively related to their predator (cf. Fig. 3). The positive relationships between the zooplankton species could have been due to some external confounding factors not considered in the analyses. However, the strong negative relationship between the predators and the large prey species, and the fact that the same pattern was observed in both zooplankton groups, indicate that the result might have been related to predator switching. Prey selectivity is a conspicuous characteristic of clupeid fishes [32]–[34]. According to the switching hypothesis, high abundance of the large zooplankton species will protect the smaller species from the negative effect of predation. Accordingly, the abundance of large zooplankton by the end of the feeding season would reflect both the predation pressure in terms of fish abundance, and the degree of protection due to prey selectivity. The result would be a strong positive relationship between the two prey species. Despite the fact that herring and sprat have an overlapping diet [33] and co-occur in high density especially in the southern North Sea during winter [27], the present study indicate that they, on a year to year scale, impacted different parts of the North Sea zooplankton community. While sprat was related to a southern and neritic zooplankton group (*C. helgolandicus* and *Para/Pseudocalanus*), herring was related to a more northern and Atlantic group (krill and *C. finmarchicus*). In sum, the results suggest that the fluctuations in the stocks of herring and sprat have contributed to the observed shifts in the zooplankton community in the North Sea. However, to explain the mechanisms involved, more detailed studies of seasonal and yearly dynamics in predator-prey interactions between zooplankton and clupeids are needed.

Although sea surface temperature was an important factor in the analyses of the original data, this relationship largely disappeared after detrending. This result stands in contrast to the findings of a number of other studies from the North Sea (see e.g. [2], [21]–[23], [26], [40]. We believe that this discrepancy might be due to several differences in data handling and analyses. First, although herring and sprat have been shown to have large effects on zooplankton in the Baltic Sea [15], [17], few studies have explicitly considered the numerical effect of these fishes on the zooplankton community in the North Sea (but see [25]. Second, earlier studies have used the cumulative abundance throughout the year as an estimate of yearly zooplankton abundance. However, in shelf ecosystems at high latitudes, the zooplankton biomass typically varies by several orders of magnitude seasonally [41]. Predation rates are generally highest during spring and summer [33]. In order to disentangle the various trophic and interspecific interactions it is necessary to consider the resulting abundance after the main interactions have taken place. Thus, in our case, we believe that it is correct to use winter abundance of zooplankton. Finally, most of the data series considered showed strong temporal trends. For obvious reasons, this can lead to spurious correlations between ocean climate and the abundance of the different species groups. Thus, although we cannot exclude climate as an important factor in regulating the zooplankton community in the North Sea, the evidence for climate impact in our analyses were largely based on trends in the dataseries.

In this study we therefore present an alternative to the predominant view of the North Sea as a bottom-up regulated and climate perturbed ecosystem. Our analyses indicate a “wasp-waist” regulation where clupeid fishes have a central position. Similar to other northern shelf ecosystems this dominant position is probably linked to harvesting and removal of major top predators from the system [5], [15]. Thus, the change in view is an important one, because it involves fishing as a more important driver of the system than previously anticipated.

## Materials and Methods

### Pelagic schooling fish

Herring (*Clupea harengus*) and sprat (*Sprattus sprattus*) are planktivorous, clupeid fishes, and major predators on copepods, euphausiids and amphipods [32], [33], [42], [43]. They are selective and opportunistic feeders, selecting the larger food items [32], [33], [43]. Herring do also switch to filter-feeding under high concentration of small food items [33]. Sprat and herring have overlapping diet when they co-occur [32], [33], suggesting that exploitation competition can occur. This might be the case in the southern and eastern part of the North Sea where high density of juvenile sprat and herring is found, especially during winter [27]. In the Baltic Sea, herring and sprat have been suggested to impact the abundance of zooplankton [44], [45] and the demography of seabirds [46]. Sprat is a small (<18 cm) pelagic schooling fish with a short life span (<5 years). In the North Sea, it is harvested in an industrial trawl fishery with huge variations in catches over the last 30 years [19]. Herring is a larger species (<30 cm) with a longer life span (<10 years). Historically, North Sea herring has been the target of an important European fishery [19], [47]. The stock has shown huge fluctuations the last 50 years. Sandeel (*Ammodytes marinus*) is another important schooling fish species in the North Sea [48]. Reliable time series on sandeel was not available, and it was therefore not included in the present study. It should be noted that this species is mainly inactive and buried in the substrate during winter. Sandeel is therefore probably not an important prey item for seabirds during winter.

We used data from the International Bottom Trawl Survey (IBTS) to analyze the abundance of sprat and herring. Data were obtained from the DATRAS (DAtabase TRAwl Surveys) database operated by the International Council for the Exploration of the Seas (ICES) (www.ices.dk). The North Sea IBTS data are described in detail in [49]. The IBTS consists of a number of standardized national research surveys. In the North Sea, the IBTS started in the 1960s and was mainly directed towards young herring. The area surveyed is shallow and both pelagic and benthic species are sampled. The longest and most comprehensive data set is from the winter survey (from the end of January to the beginning of March) each year. In the early years, the survey was restricted to the central and southern parts of the North Sea. The extent of the survey increased in the 1970s to cover the entire North Sea except for the deeper parts of the Norwegian trench. In the present study we used data from the winter survey from 1966 to 2008. Trawl haul was used as sampling unit in the analyses. See Table S1 for yearly sample size and Fig. S2 for data coverage. CPUE (Catch Per Unit Effort; number of fish caught per hour of trawling) was used as a proxy for the density of herring and sprat respectively. In the 1960s and 70s several types of fishing gears were used by the different participants in the IBTS survey. However, fishing gear became more and more standardized, and from 1983 all participants used the 36/47 Grande Ouverture Verticale (GOV) trawl. The catchability depends on fishing gear, and to control for this we included the type of fishing gear when modeling yearly abundance. We restricted the analyses to the three most frequently used types of fishing gear; GOV (11 892 trawl hauls), DHT (Deutch Herring Trawl) (964 trawl hauls) and H18 (874 trawl hauls). Catchability also varies among species and size classes [50]. With respect to herring, the IBTS survey catches mainly juvenile herring (1–2 years; 10–20 cm) and mature herring (>2 years; 20–30 cm) (Fig. S3). Juvenile herring is probably more important than mature herring as a food item for seabirds. Both juvenile and mature herring consume euphausiids and copepods in the North Sea [42]. For simplicity, we decided not to divide the data of herring into different size classes. Compared to herring, the length distribution of sprat was more homogeneous and dominated by smaller size classes (95% of catches between 5–14 cm; Fig. S3), reflecting the smaller size, the shorter life-span and the dominance of young fishes (1–2 years) in the stock.

### Zooplankton

Copepods form the major part of the mesozooplankton community of the North Sea. *Pseudocalanus elongatus*, *Paracalanus parvus*, *Microcalanus pusillus*, *Oithona similis*, *Acartia* spp., *Temora longicornis*, *Calanus finmarchicus* and *C. helgolandicus* are among the dominating species groups [41]. Changes in the community of calanoid copepods over the last 50 years has been attributed to a regime shift caused by hydro-climatic forcing from a cold period (1962–1982) to a warm period (1984–1999) [24]. In particular, there has been a shift in the dominance of the two important *Calanus* species from a dominance of the boreal *C. finmarchicus* to a dominance of the temperate *C. helgolandicus* [21]. The diet of herring in the North Sea varies by season and year, but is dominated by Euphausiids, *Calanus* spp. and *Temora* spp. [42]. The diet of sprat and herring in the Baltic Sea is dominated by *Pseudocalanus* sp., *Temora longicornis* and *Acartia* spp. [33]. As well as being related to the seasonal dynamics of zooplankton [42], the diet of sprat and herring is probably also related to the geographic gradient in the zooplankton community. In the northern North Sea, the zooplankton community is dominated by *C. finmarchicus* and krill while the southern and eastern part is dominated by *C. helgolandicus*, *Pseudocalanus* spp. and *Temora* spp. [41]. To cover the geographic gradient from north to south, potentially important prey species for sprat and herring, and different size classes of prey, we included 3 groups of copepods; *Paracalanus* spp. and *Pseudocalanus* spp. (hereafter termed *Para/Pseudocalanus*), *C. helgolandicus* (stages CV-CVI) and *C. finmarchicus* (stages CV-CVI). In addition we included krill *Euphausiacea* spp. (juveniles and adults) dominated by *Meganyctiphanes norvegica* [51]. The *Para/Pseudocalanus* group has the smallest individuals with an average size of 0.70 mm. The calanoids are much larger with sizes of 2.68 mm (*C. helgolandicus*) and 2.70 mm (*C. finmarchicus*) [52]. Krill is the group with the largest individuals (>1 cm).

We used data from the Continuous Plankton Recorder (CPR) survey from the winter period (October through February) from 1966 to 2007. Data were provided by the Sir Alister Hardy foundation. A detailed description of the sampling routine is provided by [52]. The CPR is a high-speed sampler that is towed behind merchant ships on their routine, monthly trading routes. The data covered the entire North Sea (see Fig. S2), and the coverage differed little from year to year. The device filters seawater at a depth of 7 to 9 m on a moving band of silk. After each tow the silk is divided into samples where each sample represents approximately 10 nautical miles (18 520 m) of towing and 3 m^3^ of filtered seawater. Each sample is counted with respect to plankton and the samples are positioned and dated [52]. CPR data has previously been used to map the species composition, numerical abundance and population dynamics of euphausiids in the North Atlantic and the North Sea [51], [53]–[55]. *Para/Pseudocalanus* was counted by microscope on 1/50 of each sample. Krill and the *Calanus* species were counted by eye on the entire sample. Average number of specimens per sample was: *Para/Pseudocalanus*: 67.6, *C. helgolandicus*: 3.3, *C. finmarchicus*: 3.0 and krill: 2.1.

### Seabirds

Many populations of breeding seabirds in the North Sea increased during the 1970s–80s, and subsequently declined during the two last decades [56], [57]. Changes in population size and demography monitored in breeding colonies, have been related to changes in the stocks of major prey items such as sandeel [48] and herring [1], changes in climate [40] and discards from fisheries [58]. Herring is an important food item for seabirds in the North Sea [1], [59], and sprat is a principal food item for seabirds in the Baltic Sea [46]. We therefore expected sprat and herring to be important prey species for wintering seabirds in the North Sea. Little is however known about the species specific diet of seabirds during winter [60], and in the present study we therefore selected the 10 most abundant pelagic species encountered during the winter surveys: common murre (*Uria aalge*), razorbill (*Alca torda*), little auk (*Alle alle*), Atlantic puffin (*Fratercula arctica*), Northern gannet (*Morus bassanus*), Northern fulmar (*Fulmarus glacialis*), black-legged kittiwake (*Rissa tridactyla*), herring gull (*Larus argentatus*), great black-backed gull (*Larus marinus*) and common gull (*Larus canus*).

We used data from the European Seabird at Sea (ESAS) database from the winter period (1 October–31 March) from 1981 to 1999. Data were collected by a standardized strip transect methodology [61]. Birds were counted from 6–10 m above sea level from ships steaming at a constant speed of ca. 20 km/h. All birds seen within an arc of 300 m from directly ahead to 90° to one side of the ship were counted. The surveys had a total length of 148 269 km. In total, the surveys covered the entire North Sea however, the coverage differed among years (see Table S1, Fig. S2). Following continuous transects chronologically, the counts of each seabird species were summed up along 20 km long strips. The encounter rate with seabirds (number of birds counted per kilometer) on each strip was used as sampling unit. Due to different behavior, size and coloration, different seabird species varies in detectablility. Specifically, small diving auks were probably under-estimated while gulls and fulmars that tend to follow the ship were over-estimated. The detectability of seabirds will also depend on factors such as distance from the transect line, observer, type of vessel and weather conditions. Variable practice with respect to the recording of these variables in the database made it impossible to control for them without discarding a large amount of data. We assumed that the error due to detectability was equally distributed among years and areas. It should be noted that the abundance estimates reported are relative values.

### Ocean climate

Recent studies suggest that annual averaged Sea Surface Temperature (SST) is a major climate variable that explains a large part of the ecosystem dynamics in the North Sea [23], [28]. Accordingly, we used the time series of SST to control for the effect of ocean climate in the analyses. We used the annual averaged SST for the North Sea from the COADS 1-degree enhanced dataset provided by the Research Data Archive (RDA) maintained by the Computational and Information Systems Laboratory (CISL) at the National Center for Atmospheric Research (NCAR).

## Analysis

### Abundance estimates

Time series on yearly abundance of seabirds, clupeids and zooplankton were generated by fitting the density data for each individual species to a statistical model that estimated yearly abundance, average spatial distribution and fishing gear (clupeids only) [38]. Note that the models did not estimate changes in the spatial distribution among years; that is the interaction between year and spatial distribution. Thus, the abundance estimates were sensitive to a representative sampling of the study area, since a combination of large scale changes in the spatial distribution and non-representative sampling in one year would bias the abundance estimate. Non-representative sampling was a problem for the IBTS data during the first six years (1966–71) when the sampling was concentrated in the central part of the North Sea, and for the seabird data in 1981, 83, 97, 98 and 99 when the sampling was mainly concentrated along the coast of the southern and western part of the study area. Spatial analyses of the residuals did, however, not reveal any strong trends among years in any of the species groups [38], suggesting that large scale changes in the spatial distribution was a minor problem.

The sample units defined for each dataset i.e. fish trawl hauls for clupeids, 10 nautical miles of towing for zooplankton and 20 km of observation for seabirds, were used as input to the statistical models. Because the datasets included an excess of zeroes, we decided to use a two-stage modeling approach [62]. First, presence/absence was modeled with a binomial distribution. Second, the counts of individuals conditional on presence, was modeled with a Gamma distribution [63]. We used Generalized Additive Models (GAM) using the “mgcv” library [64] in R v.2.10.1 [65] to model the count data from each species group. Average spatial distribution was modeled with three geographically fixed covariates: the geographical position in the *x* (west - east) and *y* (south – north) direction, bottom depth (*d*) and distance from coast (*c*). Geographic position was modeled with a two-dimensional smooth function; *g(x,y)*. *d* and *c* were modeled with a one-dimensional smooth function; *s(•)*. We used tensor product smooths with cubic regression spline as basis. The optimal degree of smoothing was defined by Generalized Cross Validation (GCV). Year (*A*) and fishing gear (*F*) were modeled as categorical variables. Due to variable transect lengths, log_e_(transect length) was included as an offset in the analyses of seabirds. First, the probability of counts larger than zero (*p*) was modeled using a logit link with a binomial distribution:
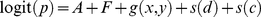
(1)Second, the count *n* given the presence of a non-zero count, was modeled using a log_e_ link with a Gamma distribution:
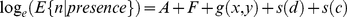
(2)where *E* is expectation.

Based on the fitted models, we used the “predict” function in the “mgcv” library to predict the average spatial distribution on a 10×10 km^2^ grid covering the entire study area in each year. Accordingly, the predicted probability of a non-zero count 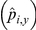 in grid cell (*i*) and year (*y*) was derived from the binomial model (eq.1). Similarly, the expected count when present 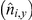 was predicted from the Gamma-model (eq. 2). The predicted count in a grid cell is then given by 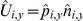 [66]. Predicted yearly abundance was accordingly calculated as; 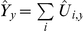. A summary of the two-stage models used to estimate yearly abundances is shown in Table S2. To reduce heterogeneity and approach normality in the residuals, the yearly abundance estimates were log_10_ transformed prior to the subsequent time series analyses.

### Detrending

Several of the time series had a temporal trend. Statistical inference drawn from analyses of non-stationary time-series might be problematic [67]. To investigate whether temporal trends in the dataseries could influence the results, trends were removed by fitting the series to GAM-functions using year as a covariate. Year was modeled with a smooth function using a thin plate regression spline as basis [64], and the residuals were used in the re-analysis of the data [67]. Because we were only interested in removing linear or curvilinear trends, we set the basis dimension of the spline equal to three. Results from analyses of both detrended and original data are presented.

### Time series analyses

We investigated how the yearly abundance estimates of seabirds and zooplankton were related to ocean climate and trophic interactions by linear models. The models were constructed according to the “wasp-waist” hypothesis, where we expected a bottom-up interaction from pelagic fish to seabirds and a top-down interaction from pelagic fish to zooplankton. The abundance of seabirds and zooplankton were accordingly used as response variables in separate analyses. As predictor variables we used SST and the abundance of herring and sprat. In addition, we investigated possible interspecific interactions between the different zooplankton groups by including the abundance estimates of the other species as predictors. To investigate how trends in the dataseries affected the estimated responses, we analyzed the original and detrended dataseries separately.

Each model was first fitted with all covariates and an AR-1 term (Auto-Regressive model of order 1) using the *gls* function in the *nlme* library in R [68]. Model simplification was done according to [69], using a backward model selection procedure. Models were compared by likelihood ratio tests or F-tests. First, we tested the full model with and without the AR-1 term using REML estimation. If the AR-1 term contributed significantly (*P*<0.05) to the model, we kept the AR-1 term, and continued the backward selection procedure using the *gls* function with ML estimation. Otherwise, we removed the AR-1 term, and proceeded with ordinary linear regression using the lm function in R. Backward selection of covariates was done by removing each covariate with the lowest fit successively until all terms contributed significantly to the model. The final model was checked for autocorrelation by including (or excluding) an AR-1 term.

Winter abundance of zooplankton was expected to be related to predation the previous summer. The abundance of clupeids, measured the previous winter was accordingly assumed to be the best proxy for predation pressure, and this time lag was used in all analyses of zooplankton. Seabirds could potentially respond to the abundance of clupeids the same winter or the abundance the previous winter. Separate models, with and without a time lag with respect to clupeids were therefore constructed. SST from the previous year was expected to have the strongest impact on zooplankton and seabirds the following winter, and this time lag was used in all analyses.

## Supporting Information

Figure S1 **Time series of the winter abundance of 10 different seabird species in the North Sea.**
(TIF)

Figure S2 **Data coverage.** A) seabirds (1981–1999), B) clupeids (1966–2008) and C) zooplankton (1966–2007).
(TIF)

Figure S3 **Length-frequency distribution of herring and sprat caught in the IBTS survey in February from 1980–2002.**
(TIF)

Table S1 **Sample size of count data on seabirds, clupeids and zooplankton**.
(DOC)

Table S2 **Summary of models used to estimate yearly winter abundance.** Two-stage GAM models relating the count of each species group to year, bottom depth, distance to coast, geographical position and fishing gear (fish only).
(DOC)
