# Supplementary figures and images for: Wasp-Waist Interactions in the North Sea Ecosystem
Source: PLoS One. 2011 Jul 28;6(7):e22729. doi: 10.1371/journal.pone.0022729 (PMC3145753; doi:10.1371/journal.pone.0022729)

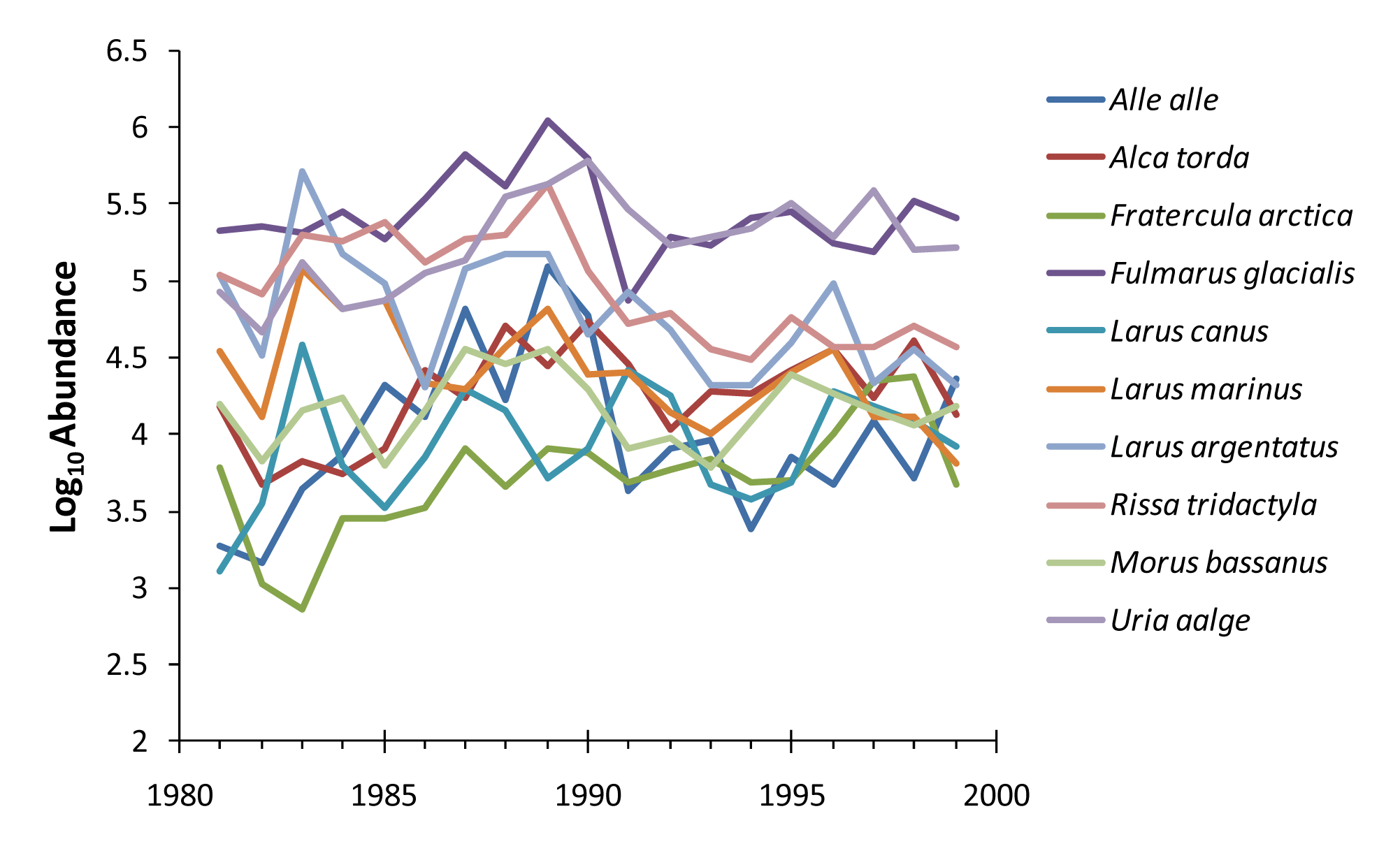

Supplement: Figure S1 — Time series of the winter abundance of 10 different seabird species in the North Sea. (TIF) [file pone.0022729.s001.tif]

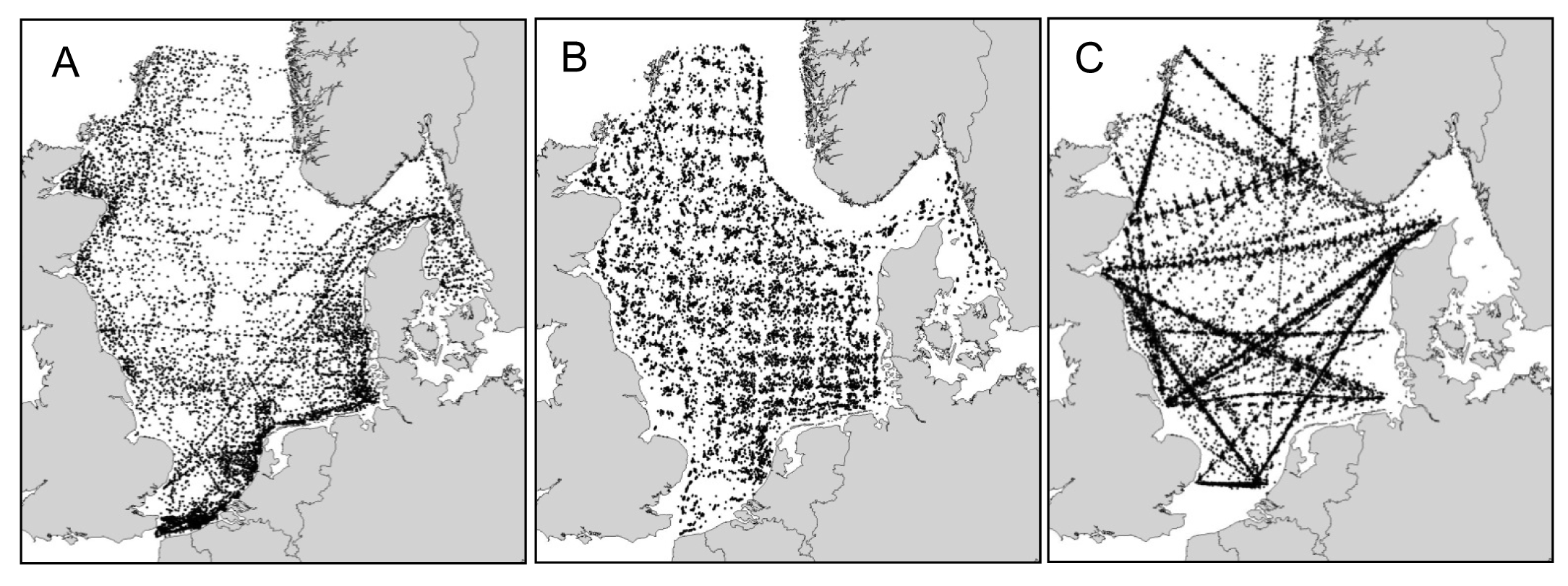

Supplement: Figure S2 — Data coverage. A) seabirds (1981–1999), B) clupeids (1966–2008) and C) zooplankton (1966–2007). (TIF) [file pone.0022729.s002.tif]

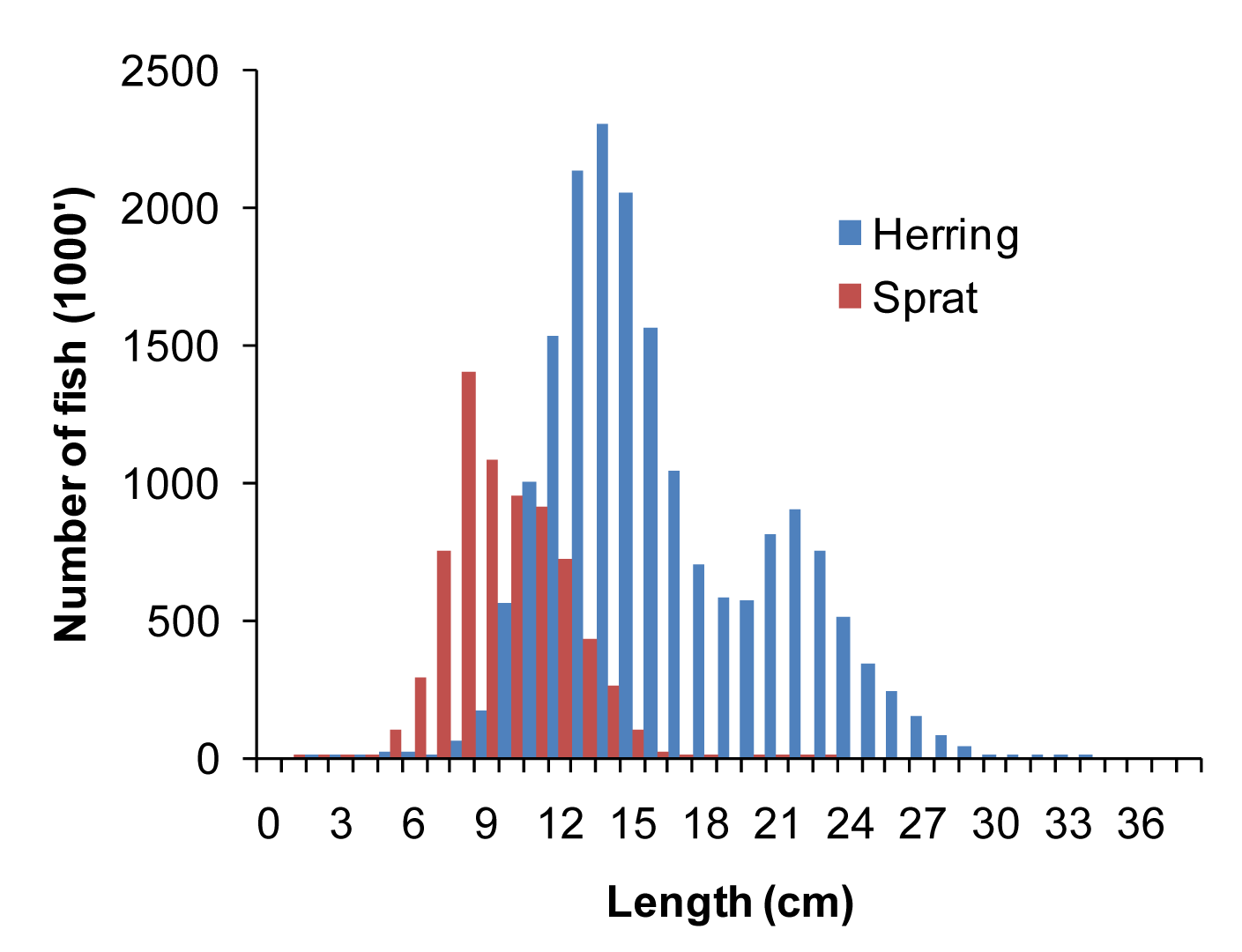

Supplement: Figure S3 — Length-frequency distribution of herring and sprat caught in the IBTS survey in February from 1980–2002. (TIF) [file pone.0022729.s003.tif]
