# Supplementary material for: Wasp-Waist Interactions in the North Sea Ecosystem
Source: PLoS One. 2011 Jul 28;6(7):e22729. doi: 10.1371/journal.pone.0022729 (PMC3145753; doi:10.1371/journal.pone.0022729)
Supplement: Table S1 — Sample size of count data on seabirds, clupeids and zooplankton. (DOC) [file pone.0022729.s004.doc]

**Table S1:** Sample size of count data on seabirds, clupeids and zooplankton from each year.

|  | Year | 1966 | 1967 | 1968 | 1969 | 1970 | 1971 | 1972 | 1973 | 1974 | 1975 | 1976 |
| --- | --- | --- | --- | --- | --- | --- | --- | --- | --- | --- | --- | --- |
| Sample size | Seabirds |  |  |  |  |  |  |  |  |  |  |  |
|  | Clupeids | 87 | 119 | 135 | 107 | 129 | 141 | 154 | 151 | 154 | 224 | 202 |
|  | Zooplankon | 463 | 426 | 498 | 582 | 430 | 481 | 503 | 445 | 319 | 326 | 385 |
|  |  |  |  |  |  |  |  |  |  |  |  |  |
|  | Year | 1977 | 1978 | 1979 | 1980 | 1981 | 1982 | 1983 | 1984 | 1985 | 1986 | 1987 |
| Sample size | Seabirds |  |  |  |  | 174 | 779 | 129 | 219 | 332 | 363 | 348 |
|  | Clupeids | 222 | 324 | 329 | 381 | 306 | 329 | 426 | 452 | 496 | 510 | 525 |
|  | Zooplankon | 440 | 319 | 255 | 265 | 481 | 344 | 403 | 300 | 491 | 329 | 449 |
|  |  |  |  |  |  |  |  |  |  |  |  |  |
|  | Year | 1988 | 1989 | 1990 | 1991 | 1992 | 1993 | 1994 | 1995 | 1996 | 1997 | 1998 |
| Sample size | Seabirds | 1057 | 872 | 612 | 535 | 673 | 1114 | 537 | 688 | 278 | 167 | 477 |
|  | Clupeids | 391 | 415 | 371 | 409 | 367 | 361 | 356 | 331 | 318 | 354 | 391 |
|  | Zooplankon | 378 | 393 | 410 | 378 | 365 | 515 | 443 | 435 | 480 | 501 | 472 |
|  |  |  |  |  |  |  |  |  |  |  |  |  |
|  | Year | 1999 | 2000 | 2001 | 2002 | 2003 | 2004 | 2005 | 2006 | 2007 | 2008 |  |
| Sample size | Seabirds | 255 |  |  |  |  |  |  |  |  |  |  |
|  | Clupeids | 351 | 369 | 416 | 414 | 403 | 362 | 377 | 369 | 345 | 357 |  |
|  | Zooplankon | 470 | 481 | 529 | 511 | 509 | 477 | 460 | 533 | 199 |  |  |
