# Supplementary material for: Wasp-Waist Interactions in the North Sea Ecosystem
Source: PLoS One. 2011 Jul 28;6(7):e22729. doi: 10.1371/journal.pone.0022729 (PMC3145753; doi:10.1371/journal.pone.0022729)
Supplement: Table S2 — Summary of models used to estimate yearly winter abundance. Two-stage GAM models relating the count of each species group to year, bottom depth, distance to coast, geographical position and fishing gear (fish only). (DOC) [file pone.0022729.s005.doc]

**Table S2.** Summary of two-stage models relating the count of each species group to *year*, bottom *depth*, distance to coast (*dist*), geographical position (*g(X,Y)*) and fishing *gear* (fish only). Number of observations (*n*), percent deviance explained (*%dev*) for each model, and estimated degrees of freedom (*edf*) for each variable are given

|  |  |  |  | Parametric terms | | | |  | Smooth terms | | | | | |
| --- | --- | --- | --- | --- | --- | --- | --- | --- | --- | --- | --- | --- | --- | --- |
|  |  |  | % | Year | | Gear | |  | g(X,Y) | | s(dist) | | s(depth) | |
|  |  | *n* | *dev* | *df* | *P* | *df* | *P* |  | *edf* | *P* | *edf* | *P* | *edf* | *P* |
| Little auk | 1) | 9609 | 33 | 18 | *** |  |  |  | 19.5 | *** | 2.1 | *** | 1.0 | NS |
|  | 2) | 764 | 47 | 18 | *** |  |  |  | 19.1 | *** | 1.0 | * | 1.0 | NS |
| Puffin | 1) | 9609 | 33 | 18 | *** |  |  |  | 18.5 | *** | 3.7 | *** | 1.9 | NS |
|  | 2) | 725 | 36 | 18 | *** |  |  |  | 14.9 | *** | 3.6 | NS | 8.8 | ** |
| Common murre | 1) | 9609 | 9 | 18 | *** |  |  |  | 20.6 | *** | 3.4 | *** | 3.4 | *** |
|  | 2) | 5960 | 24 | 18 | *** |  |  |  | 21.8 | *** | 2.8 | ** | 3.7 | *** |
| Razorbill | 1) | 9609 | 14 | 18 | *** |  |  |  | 19.9 | *** | 3.3 | ** | 3.3 | *** |
|  | 2) | 2017 | 59 | 18 | *** |  |  |  | 22.6 | *** | 3.9 | ** | 3.7 | *** |
| Gannet | 1) | 9609 | 18 | 18 | *** |  |  |  | 19.9 | *** | 3.1 | *** | 3.9 | *** |
|  | 2) | 2149 | 26 | 18 | *** |  |  |  | 21.4 | *** | 2.7 | NS | 3.5 | *** |
| Fulmar | 1) | 9609 | 32 | 18 | *** |  |  |  | 20.1 | *** | 2.4 | *** | 3.5 | *** |
|  | 2) | 3793 | 32 | 18 | *** |  |  |  | 23.4 | *** | 3.6 | ** | 4.0 | *** |
| Kittiwake | 1) | 9609 | 5 | 18 | *** |  |  |  | 15.6 | *** | 2.6 | *** | 3.8 | *** |
|  | 2) | 4534 | 21 | 18 | *** |  |  |  | 20.8 | *** | 3.9 | *** | 3.9 | *** |
| G. black-b. gull | 1) | 9609 | 9 | 18 | *** |  |  |  | 21.2 | *** | 3.5 | *** | 3.9 | *** |
|  | 2) | 3548 | 20 | 18 | *** |  |  |  | 19.8 | *** | 3.9 | * | 2.0 | *** |
| Herring gull | 1) | 9609 | 12 | 18 | *** |  |  |  | 21.7 | *** | 3.9 | *** | 4.0 | *** |
|  | 2) | 3844 | 22 | 18 | *** |  |  |  | 20.2 | *** | 3.9 | *** | 3.6 | *** |
| Common gull | 1) | 9609 | 32 | 18 | *** |  |  |  | 23.7 | *** | 3.3 | *** | 1.0 | NS |
|  | 2) | 2256 | 23 | 18 | *** |  |  |  | 20.3 | *** | 2.6 | ** | 3.8 | ** |
| Herring | 1) | 13730 | 18 | 42 | *** | 2 | ** |  | 21.7 | *** | 3.9 | *** | 3.8 | *** |
|  | 2) | 11485 | 21 | 42 | *** | 2 | ** |  | 23.9 | *** | 3.8 | *** | 3.9 | *** |
| Sprat | 1) | 13730 | 44 | 42 | *** | 2 | *** |  | 22.0 | *** | 3.8 | *** | 3.4 | *** |
|  | 2) | 8178 | 30 | 42 | *** | 2 | *** |  | 23.9 | *** | 3.9 | *** | 2.8 | *** |
| Krill | 1) | 17873 | 15 | 41 | *** |  |  |  | 22.7 | *** | 1.0 | ** | 3.0 | *** |
|  | 2) | 6775 | 22 | 41 | *** |  |  |  | 22.3 | *** | 3.8 | * | 3.2 | *** |
| *C. finmarchicus* | 1) | 17873 | 20 | 41 | *** |  |  |  | 22.8 | *** | 2.9 | *** | 3.0 | ** |
|  | 2) | 5447 | 25 | 41 | *** |  |  |  | 22.4 | *** | 3.3 | *** | 1.0 | ** |
| *C. helgolandicus* | 1) | 17873 | 11 | 41 | *** |  |  |  | 19.4 | *** | 2.4 | * | 3.7 | NS |
|  | 2) | 8237 | 11 | 41 | *** |  |  |  | 22.6 | *** | 3.7 | *** | 3.6 | * |
| *Para/pseudoc.* | 1) | 17873 | 10 | 41 | *** |  |  |  | 22.8 | *** | 1.0 | NS | 2.3 | ** |
|  | 2) | 6567 | 14 | 41 | *** |  |  |  | 20.3 | *** | 1.0 | NS | 3.9 | *** |

*** *P*<0.001, ** 0.001<*P*<0.01, * 0.01<*P*<0.05, NS *P*>0.05

1) *Logistic models*: Generalized Additive Models (GAMs) of the presence of individuals using a logit link with a binomial distribution

2) *Gamma models*: GAMs of the counts conditional on presence, using a log_e_ link with a Gamma distribution.
